# Toward a Mouse Neuroethology in the Laboratory Environment

**DOI:** 10.1371/journal.pone.0011359

**Published:** 2010-06-29

**Authors:** Anthony M. Oliva, Ernesto Salcedo, Jennifer L. Hellier, Xuan Ly, Kanthaiah Koka, Daniel J. Tollin, Diego Restrepo

**Affiliations:** 1 Department of Cell and Developmental Biology, University of Colorado Medical School, Aurora, Colorado, United States of America; 2 Neuroscience Program, University of Colorado Medical School, Aurora, Colorado, United States of America; 3 Rocky Mountain Taste and Smell Center, University of Colorado Medical School, Aurora, Colorado, United States of America; 4 Department of Physiology and Biophysics, University of Colorado Medical School, Aurora, Colorado, United States of America; University of Maryland, United States of America

## Abstract

In this report we demonstrate that differences in cage type brought unexpected effects on aggressive behavior and neuroanatomical features of the mouse olfactory bulb. A careful characterization of two cage types, including a comparison of the auditory and temperature environments, coupled with a demonstration that naris occlusion abolishes the neuroanatomical changes, lead us to conclude that a likely important factor mediating the phenotypic changes we find is the olfactory environment of the two cages. We infer that seemingly innocuous changes in cage environment can affect sensory input relevant to mice and elicit profound effects on neural output. Study of the neural mechanisms underlying animal behavior in the laboratory environment should be broadened to include neuroethological approaches to examine how the laboratory environment (beyond animal well-being and enrichment) influences neural systems and behavior.

## Introduction

Neuroscientists use laboratory experiments to study the neural basis of behavior. The effects of conditions in the laboratory environment on animal physiology and behavior have been studied extensively from enrichment, naturalistic experience, and well-being points of view [1], [2], [3], [4], [5]. However, relatively little attention is paid to how differences in “standard” housing conditions affect experiments. Although there is some evidence to the contrary [2], [3], [6], scientists often assume that there is little impact of differences in housing environment (provided there is no enrichment) on the data they acquire. We were forced to challenge these assumptions when we noticed marked changes in aggressive behavior and olfactory bulb (OB) neuroanatomy in our mice after a move to a new animal facility. Here we report a systematic comparison of the differences in olfactory glomeruli and intermale aggressive behavior in animals housed in two different cage types.

## Results

### Intermale Aggression Differs Between Mice Raised in Different Cage Environments

**Figure 1:**
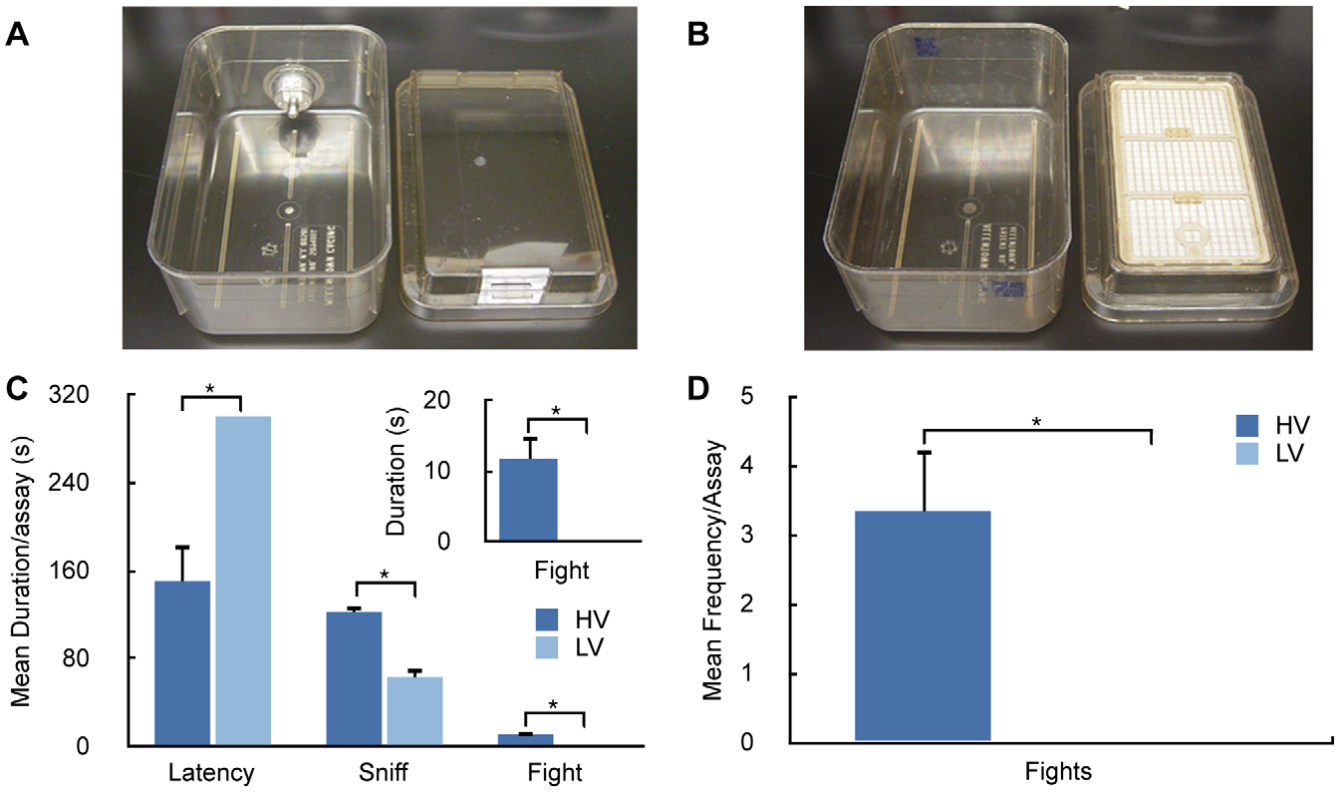
Housing differences result in marked behavioral changes. **A.** Picture of a high ventilation (HV) cage and **B.** a low ventilation (LV) cage. The duration (**C**) and frequency (**D**) of various types of aggressive behavior are significantly different depending on the type of cage (HV or LV) the mice were housed in. Resident males were exposed to an intruder male for five minutes. A mixed ANOVA revealed a significant effect of cage type on the latency to first fight (Latency) (F_1,12_ = 7.09, P = 0.0027), the total time spent interacting (Sniff) (F_1,12_ = 21.56, P = 0.0006), the total time spent fighting (Fight) (F_1,12_ = 11.35, P = 0.0039), and on the number of fights (Fights) (F_1,12_ = 13.33, P = 0.0022). When mice did not attack, the latency was set to 300 sec. The bars represent mean±SEM (n = 6 per group).

Here we studied differences in intermale aggression in two different types of cages. In high ventilation cages (HV cages, Fig. 1A) air was mechanically exchanged with fresh air once every minute whereas in low ventilation cages (LV cages, Fig. 1B) air was exchanged passively through a filter in the cover. Because it is known that structural features of the OB are sensitive to the olfactory environment [7], [8] and that the olfactory system plays an essential role in aggression in mice [9], [10], [11], we hypothesized that the changes in intermale aggressive behavior could be elicited by differences in cage type. To test this hypothesis, we conducted a study of the effect of cage environment (HV vs. LV) on behavior and OB neuroanatomy. We concentrated on main olfactory bulb neuroanatomy because it is known to be modified by olfactory environment [7], [8] and the main olfactory system is involved in aggression [9].

Adult 12-week-old mice reared in the new facility in otherwise identical conditions were either kept in the HV cages or transferred to LV cages for four weeks. Figures 1C and D show clear cage-dependent differences in the aggressive behavior of resident males toward intruder males: Residents of HV cages showed significantly more interaction and aggression than residents of LV cages.

### Urine Volatile-Responsive Lateral P2 Glomeruli Differ in Size and Number in Mice Raised in Different Cage Environments

**Figure 2:**
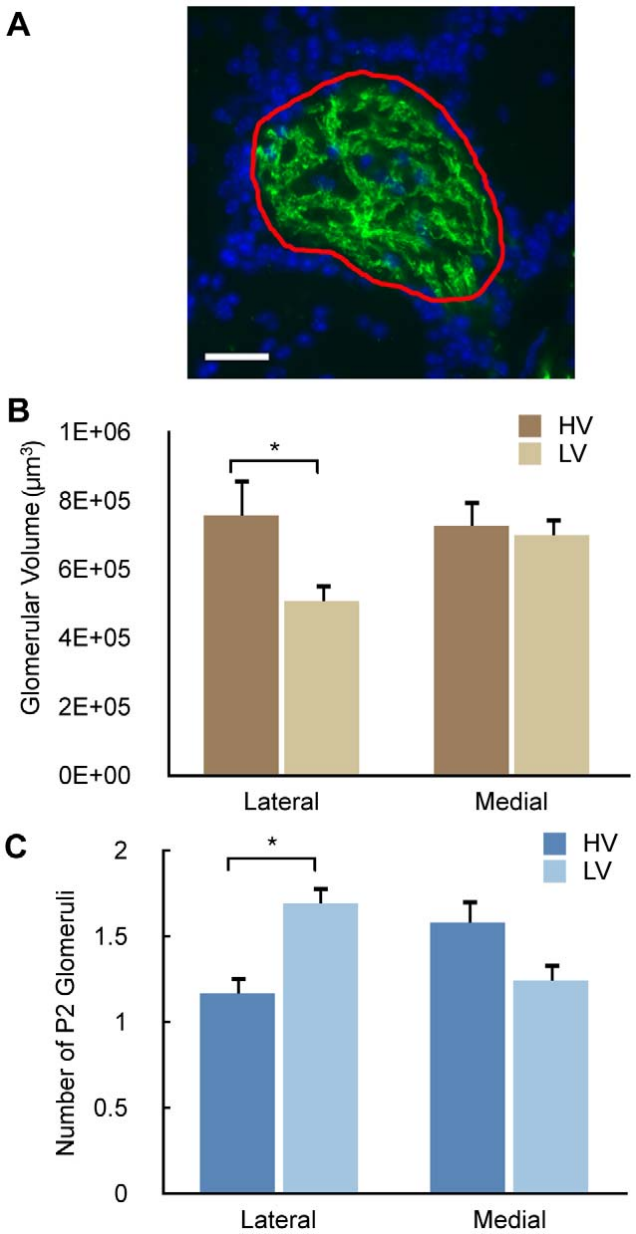
Cage environment affects the neuroanatomical characteristics of the urine volatile-responsive P2 glomerulus. **A**. Fluorescent micrograph of a representative P2 glomerulus. P2 olfactory sensory neuron axons are in green, juxtaglomerular cell nuclei (labeled with DAPI) in blue, and outline of glomerulus used for volume measurement in red. **B.** Bar graph illustrating the effect of odor environment on P2 glomerular volume in the lateral and medial domains. A mixed effects ANOVA indicated significant effects on volume of cage type (F_1,12_ = 6.32, P = 0.027) and of the interaction between cage type and domain (lateral vs. medial) (F_1,49_ = 10.9, P = 0.0018). Post-hoc tests revealed a significant effect of environment on the lateral glomerular volume (P = 0.0013) whereas the medial glomerular volumes did not show any differences (P = 1.0). All bar graphs are mean ± SEM (n = 8 for LV and 6 for HV cages). Asterisks indicate post-hoc tests with P<0.05. **C.** Bar graph illustrating the effect of odor environment on the number of P2 glomeruli in the lateral and medial domains. A mixed ANOVA revealed a significant effect of the interaction between environment and domain on the number of P2 glomeruli (F_1,26_ = 11.98, P = 0.0019). Post-hoc comparison of the number of lateral, but not medial, P2 glomeruli showed a significant effect of environment (P = 0.03). The data in the LV cages is reproduced with permission from a previous publication in the Journal of Comparative Neurology [15].

Olfactory cues processed by the main (and accessory) olfactory systems [9], [12] can mediate intermale aggression. Therefore, our behavioral data raise the question whether rearing the mice in the different environments affects olfactory system structure or function. To examine this question, we characterized the impact of cage environment on the neuroanatomy of glomeruli in the OB. Glomeruli receive incoming axons from olfactory sensory neurons bearing the same olfactory receptor synapse onto dendrites of OB neurons (juxtaglomerular, mitral and tufted cells) [13]. We focused on P2 glomeruli, which are found in the medial and lateral domains of the OB [14], because they are responsive to urine volatiles [15], a prominent odor in the cages and one used by mice for communication relevant to social and sexual interactions [16], [17], [18]. Interestingly, a precedent for laboratory-to-laboratory variation in P2 glomeruli has previously been reported. Depending on the reporting laboratory, the number of genetically identified P2 glomeruli per bulb in the mouse OB varies from 2 to 5 [14], [19], [20], [21], [22] despite their common source [14]. Thus, it is plausible that housing conditions affect the formation of P2 glomeruli in some as yet to be understood fashion. Concordantly, we found significant effects of cage environment on the number (Figure 2C) and volume (Figure 2B) of P2 glomeruli. Unexpectedly, cage environment affected the number and volume of *lateral* P2 glomeruli, but did not have an effect on *medial* glomeruli. In a separate experiment performed on mice that were born and housed in LV cages, we obtained glomerular volume results similar to those of the LV cage results in Fig. 2C (data not shown).

### Sensory Deprivation by Naris Occlusion Abolishes Difference in P2 Glomerular Volume and Number in Mice Reared in Different Cage Environments

**Figure 3:**
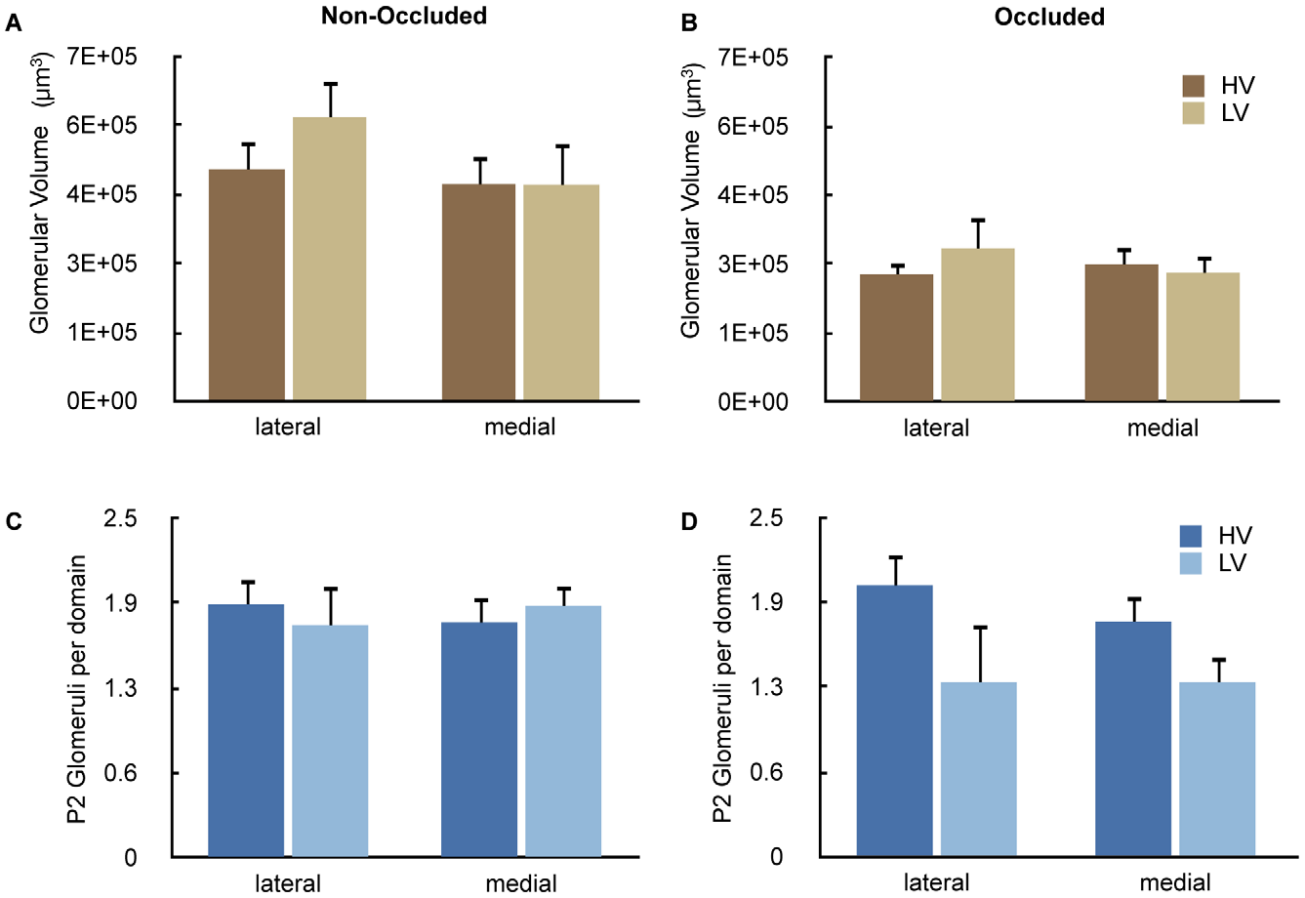
Naris occlusion abolishes the difference in glomerular volume and glomerular number between animals raised in different environments. **A and B.** Glomerular volume: **A** (non-occluded naris) and **B** (occluded naris). Occlusion of one naris abolished the difference in glomerular volume between HV and LV cages. A mixed effects ANOVA showed no differences in glomerular volume between different cage types (F_1,37_ = 0.37, P = 0.55). We did find significant differences in glomerular volume between the naris occluded and unoccluded sides (F_1,100_ = 16.7, P<0.0001). **C and D.** Number of glomeruli: C (non-occluded naris) and D (occluded naris). A mixed effects ANOVA showed no differences in glomerular number between different cage types (F_1,20_ = 1.82, P = 0.19) and between occluded and unoccluded sides. The data in the LV cages is reproduced with permission from a previous publication in the Journal of Comparative Neurology [15].

In order to determine whether exposure of the olfactory epithelium to different environments in the two cage types affected the neuroanatomical features of the P2 glomeruli, we performed sensory deprivation by naris occlusion and placed the mice in the two types of cages for four weeks. Previous work in our laboratory showed that naris occlusion affected the volume and number of P2 glomeruli[15]. As expected, naris occlusion abolished the differences in P2 glomerular volume (Fig. 3A and B) and the number of P2 glomeruli (Fig. 3C and D) between animals kept in the two types of cages (Fig. 3). These experiments suggest that exposure of the olfactory epithelium to volatiles in the cage environment accounts for the differences in neuroanatomical features for P2 glomeruli in the different cage types.

### Corticosterone Levels do not Differ Between Mice Raised in Different Cage Environments

Corticosterone, a precursor to aldosterone, is a glucorcorticoid produced by the adrenal cortex in response to ACTH (corticotropic hormone). Glucorcorticoid production increases in response to stress making corticosterone a useful biomarker of stress [23], [24]. To test whether mice in the two different styled cages experienced different levels of stress, we measured corticosterone levels in adult mice singly housed in LV or HV cages after four weeks. We found no significant difference in the corticosterone levels between the two groups of mice. LV mice had an average of 48.32±4.94 pg/mg of corticosterone in their fecal matter, while HV mice had 56.574±7.43 pg/mg (t-test, p = 0.3820).

### Temperature Differed by Less than One Degree Celsius Between Different Cage Environments

A factor affecting behavior and brain development could be a difference in temperature in the two cage environments due to the difference in airflow. We recorded the temperature of LV cages, HV cages not connected to the ventilation system (HV no ventilation cages) and in HV cages connected to the ventilation system (HV cages). The mean and corresponding SD along with the overall range of temperatures over 24 hrs in °C was 23.2°±0.26° [22.7°–23.9°] for HV no ventilation, 22.1°±0.3° [21.4°–22.6°] for LV cages, and 22.6°±0.2° [22.3°–23.9°] for HV cages. Temperature fluctuated within a tight ∼1°C range. These minor temperature fluctuations are unlikely to be the source of the results observed.

### Acoustic Factors are similar across Cage Conditions

**Figure 4:**
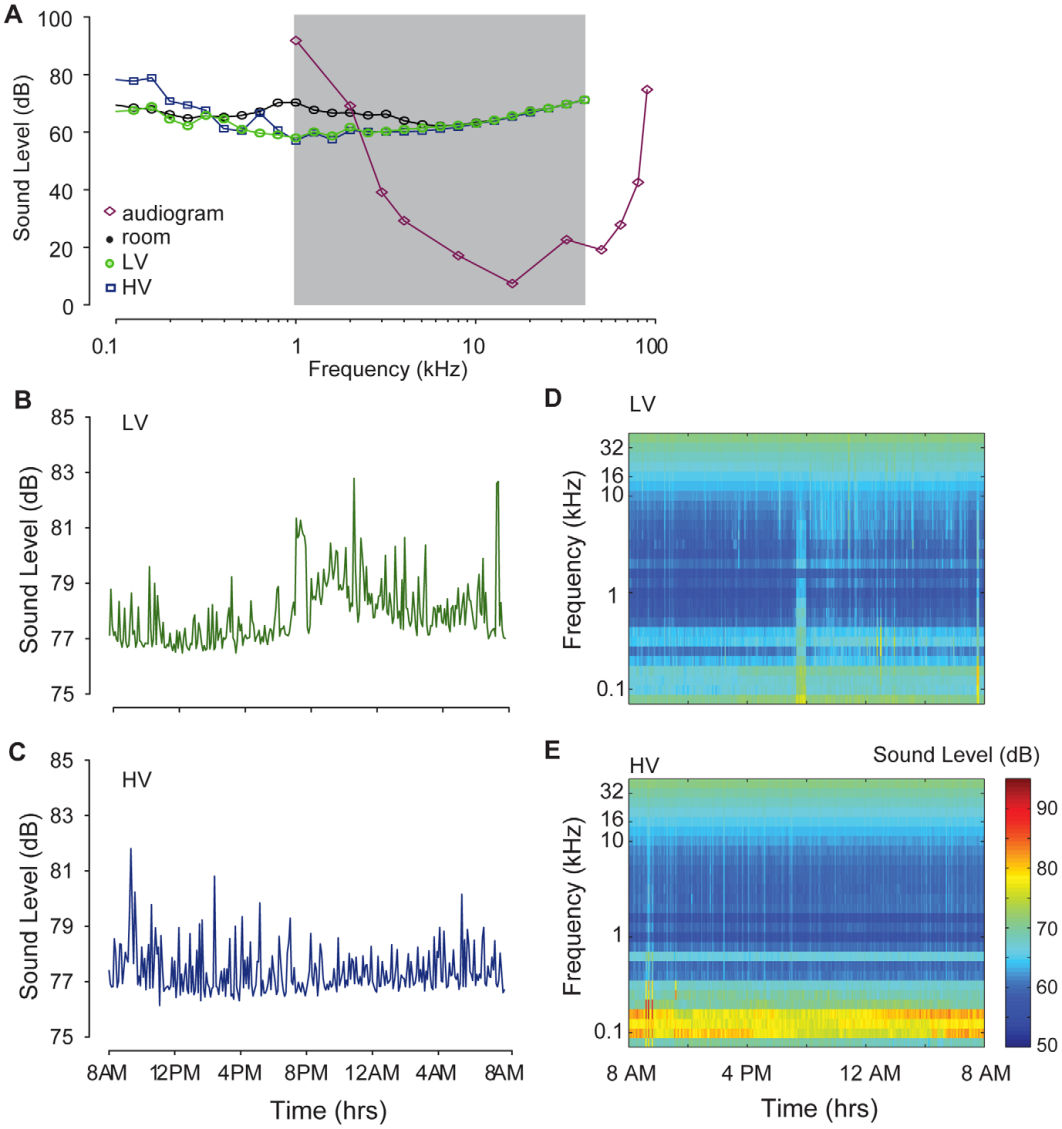
Sound levels are not affected by cage environment. **A.** Sound levels (dB) computed in 1/3-octave bands (80 Hz–40.3 kHz) for the two cage conditions: LV (green), and HV (blue). An additional acoustical measurement was taken outside the cage (room, black). The audiogram (purple) of the mouse is replotted from [25]. The shade region indicates the range of sound frequencies over which the broadband sound levels were computed in **B-C**. Broadband sound levels computed every 5 minutes over the range of frequencies indicated by the shading in **A** (1–40.3 kHz). **D-E.** Spectrograms showing sound level in 1/3-octave bands (color bar, right-hand side of panel E) measured every 5 minutes over a 24 hr period.

Fig. 4A shows representative samples of the acoustic recordings taken from the two types of cages (HV and LV) and from the ambient room. Data are plotted as equivalent dB SPL, or sound level, computed in 1/3 octave bands from 80 Hz to 40.3 kHz.). For comparison, we have also plotted the mouse audiogram [25]. The shaded grey area indicates the range of frequencies over which the broadband sound levels were computed (Fig. 4B–C). The acoustic recordings show similar spectra at all frequencies above ∼1 kHz. There were some larger differences for lower frequencies, where the HV cages had sound levels 5–10 dB more than the LV cages. As these changes occurred for frequencies <1 kHz, they would not be audible to mice (i.e., these levels fall outside the audiogram). Also, acoustic recordings outside the cages revealed sound levels that were elevated by 5–10 dB relative to the recordings in the cages for frequencies <∼8 kHz. Thus, the cages reduced the noise produced by the HVAC system but in general the mice would not be expected to hear much of this low-frequency noise. The data indicate that the ventilation rate of the cages had no appreciable impact on noise levels within the hearing range of the mouse.

Figs. 4B–C show the broadband sound levels taken every 5 minutes over 24 hrs for the two conditions. Broadband sound levels (equivalent dB SPL) were computed for frequencies from 1–40.3 kHz (Fig. 4A, shaded area). Computed over 24 hrs, the mean (±1 SD) of the sound levels along with the overall range was 78±1.0 dB [76.5–82.8 dB] for LV condition\and 77±0.8 dB [76.1–81.8 dB] for the HV conditions. Both cage conditions generally had sound levels averaging ∼77 dB, but with periods of increased levels. To put these sound levels into perspective, levels of 70 dB are approximately where most people would listen to radio or television broadcasts. 75 dB is representative of the sound level of a common vacuum cleaner or the inside of a passenger vehicle traveling at 40 mph [26]. Long-term exposure to noise levels >85 dB can do permanent damage to the auditory system and can be quite stressful [26]. The sound levels in the animal facility and on the insides of the cages are well below this level. Most of the sound levels in the animal facilities results from the low-frequency power (<1 kHz) due to HVAC ventilation fan noise with periodic interruptions from researchers and animal care staff.

Figs. 4D–E show spectrograms (sound spectra over time) over the 24h recording periods for the two ventilation conditions. In this representation, common and continuous sources of noise, such as the HVAC system, would be represented as horizontal bands of increased sound levels. For the most part, these continuous noise sources produced high sound levels only at low frequencies, below the hearing range of mice. The vertical bands of increased sound levels indicate transient acoustic disturbances, such as the presence of fellow researchers and/or animal caregivers entering and leaving the room and doing procedures. We found no evidence of persistent high-intensity, high frequency sources of noise that might be considered bothersome or stressful to mice. As the sound levels were comparable in all cage conditions, acoustic factors are unlikely to cause the behavioral and neuroanatomical differences reported in this paper.

## Discussion

The findings here demonstrate that something as seemingly innocuous as cage type can have profound effects on both neuroanatomy *and* behavior. Our experiments demonstrate that what from our perspective appears to be an inconsequential change in environment can result in a surprisingly substantial change in behavior and neuroanatomy. The measurements of auditory environment and temperature make it unlikely that these factors contributed to the changes observed. In addition, we do not see a difference in the levels of the stress biomarker corticosterone in mice housed in the two differed styled cages. However, the abolishment by naris occlusion of P2 glomerular volume differences in mice reared in different cage environments strongly suggests that the differences in the olfactory environment in the two cages (likely caused by differences in air exchange) cause neuroanatomical changes that could contribute to changes in behavior.

Differences in behavioral phenotype between laboratories have been recognized in previous studies [6], [27], [28]. For example, Wahlsten and co-workers found significant differences in behavioral phenotypes for different mouse strains between laboratories for the elevated plus maze test (strain*lab differences), but did not find strain*lab effects for ethanol preference or locomotor activity [6]. The differences in behavioral phenotype are elicited by factors such as cage enrichment [4], [5], naturalistic experience [1], cage position in the colony room [29], size of the drinking spout tube [30], and the identity of the experimenter performing the test [5], [31]. Here we show that changes in cage type result in substantial changes in intermale aggression and, for the first time, differences in the neuroanatomy of mice. Previous studies have shown that odor enrichment in the housing environment and flavors included in food during shipping of mice affect olfactory discrimination [4] and flavor preferences [32], respectively. In comparison, our study brings attention to the fact that differences in the olfactory environment of different cage styles likely cause the differences in size of olfactory glomeruli and affect intermale aggression.

Given the substantial changes in neuroanatomy and behavior we find to be resultant from cage environment, we believe that it is important to develop a neuroethology of the laboratory environment. By definition, neuroethology is the study of the neural basis of behavior under natural conditions [33], [34]. Nevertheless, we employ an unorthodox use of this term to highlight the importance of using a neuroethological approach to study the effect of environment in the animal facility on behavior and neural output. A unifying goal of the neuroethologist is to understand behavior through evolutionary explanations [35]. Neuroethologists suggest that forces of natural and sexual selection favor behaviors that maximize the reproductive success of individuals within the context of their native physical and social environments. We propose that it is advantageous to bring this approach to the understanding of how changes in cage environment affect mice. For example, evolution has clearly resulted in an important role of olfaction in communication, evaluation of potential reward and punishment, and mating in macrosmatic animals such as mice [17], [36]. As such, olfaction, and other sensory inputs that are key to a laboratory animal's behavior, should be taken into account when the living environment is modified. In addition, neuroethologists emphasize a thorough evaluation of the environment. We believe that it is important to evaluate the laboratory environment of mice within the context of the known characteristics of their sensory input (as opposed to making human-centered assumptions on how changes in cage environment will affect mice). Furthermore, it will be important to compare the behavior of mice raised in the laboratory or in a natural environment. While there has been work on how enrichment and naturalistic experience [1] transforms behavior and neuroanatomy in laboratory rodents, there is a need to understand how laboratory mice relate to their wild brethren. Relatively few neuroethological studies of natural behavior have been performed in mice [17], [36], [37].

Thus, to explore the neural basis of complex behavioral traits unencumbered by inadvertent laboratory confounds, it is advisable to develop a neuroethology of animal facilities. Such a neuroethology could be used to identify, across a wide array of disciplines, the breadth of these unidentified laboratory environment effects. Moreover, it could be used to establish a new set of standards that could address any underlying environmental confounds and thereby permit the direct comparison of results from different laboratories. Such an approach would maximize the relevance of laboratory findings to natural processes and behaviors.

## Materials and Methods

### Animals

High ventilation (HV) or individually ventilated cages were Micro-VENT cages (MBS75JHTMV) from Allentown Inc (Allentown, NJ) with air exchange of one volume per minute. Low ventilation (LV) or static micro-barrier cages were Static Micro-BARRIER cages (MBS75JHT) with passive air exchange through a Reemay™ filter medium cover. Mice had food and water available *ad libitum* and were kept under a reversed light cycle (12 noon off, 12 midnight on). Relative humidity was maintained at 30%. The bedding used was Harlan Sani-Chips (Harlan Teklad, Madison, WI). We did not notice any difference in mouse weight (16-week-old C57BL/6 mice: weight for six mice kept in LV cages was 36.5±0.43 and for six mice housed in HV cages it was 36.16±0.7, mean±SEM, p = 0.75 with a two tailed t test). All experiments were performed under protocols approved by the Animal Care and Use Committee of the University of Colorado Denver.

### Intermale Aggression

For the behavioral studies, C57/BL6 male 16-week-old mice were housed singly in HV or in LV cages for four weeks. The mice were tested in three 5-minute trials during the dark phase of their light/dark cycle when they are most active. A stranger intruder mouse was introduced into the home cage of the resident mouse and three times were measured to assess differential aggression: duration of total interactions, latency to first fight, and total fight time. Additionally, we measured the number of fights during each trial. Tests were performed on six mice per group (LV vs. HV).

### Neuroanatomical Measurements

For the anatomical portion of this study, fourteen adult male P2-IRES-*tau*GFP mice [14] were born and reared in HV cages. At 12 weeks of age, eight mice were transferred into LV cages while six remained housed in HV cages. The mice were subsequently anesthetized, perfused transcardially with 0.1M phosphate-buffered saline (PBS) followed by 0.1M PBS containing 4% paraformaldehyde. The brains were harvested and post-fixed for 2 hours on ice before cryoprotection by incubation in 0.1M PBS with 30% sucrose overnight at 4°C. The brains were placed in a positional mold and cut transversally in a cryostat at 18 µm [19]. All sections were photographed on a Nikon Eclipse E600 microscope (Tokyo, Japan) with a 40X objective using a Spot RT camera. A P2 glomerulus was defined as a region of GFP-labeled neuropil bounded by DAPI stained juxtaglomerular (JG) cells (Fig. 2A). To determine P2 glomerular volume, we used ImageJ software to calculate cross-sectional areas of a particular glomerulus in serial sections. The volume of the glomerulus was calculated by summing the cross-sectional areas and multiplying by the thickness (18 µm).

### Naris Occlusion

The surgical procedure was adapted from Baker et al. [38]. Twelve-week old adult mice were anesthetized with ketamine–xylazine (100 µg/g–20 µg/g bodyweight). The left or right naris was cauterized (Aaron Medical Industries, St. Petersburg, FL) in fourteen mice. After surgery, ointment and infant Tylenol were administered to alleviate pain. Seven of these mice were subsequently group-housed in LV cages and seven in HV cages and sacrificed 4 weeks after surgery.

### Corticosterone Measurements

A separate group of 10 male P2-IRES-*tau*GFP mice [14] were born and reared in HV cages. At 12 weeks of age, five mice were transferred into LV cages while five remained housed in HV cages. Four weeks later mouse fecal pellets were collected from the rodent's home cage one hour after the bedding had been replaced. After collection, pellets were frozen overnight at −80°C, and then shipped overnight (on dry ice) to the Cayman Chemical Company (Ann Arbor, Michigan) for assay service. At Cayman, the Cayman Corticosterone EIA Kit protocols were followed exactly. Briefly, mouse pellets were lyophilized overnight to ensure removal of all water. Each sample was divided into two parts (one part not to be spiked, and the other part to be spiked with 10,000 pg of corticosterone). 1 ml of 90% ethanol was added to each sample and samples were then homogenized using a Precellys® 24 for three twenty second cycles at 5200 rpm. Samples were spun down to obtain supernatants, which were then dried under nitrogen and each reuspended in 1 ml of EIA Buffer. A standard curve was established by serial dilution of corticosterone between 8.2 and 5,000 pg/mL using EIA Buffer as the matrix. The concentration of each sample was calculated from a logistic four-parameter fit of the standard concentrations versus % Bound/Maximum Bound (%B/B_0_). Corticosterone concentrations were normalized for recovery.

### Methods for acoustic and temperature measurements

The recording of sound level and temperature were monitored in two otherwise identical rooms in the animal facility. The no ventilation and low ventilation conditions were studied in one room and the high ventilation condition was studied in another room; a different room was required for the latter condition because the flow-rate could be easily altered in that room. Sound level and temperature were recorded in each condition for 24 hours beginning at 8AM. The microphone and temperature probe were positioned at the center of a polycarbonate mouse cage complete with bedding, food container, etc, but no animal. This test cage was in the center of a rack of cages all of which contained mice. Sound levels and temperature were recorded using a custom written MATLAB (v7.1, The Math Works Inc, Natick, MA) program which sampled the acoustic and temperature data every 5 minutes. The acoustic data were 1-sec in duration and collected at a nominal sampling rate of 100 kHz via an analogue to digital converter (RP2.1, Tucker Davis Technologies (TDT), Alachua FL). The free-field microphone (Brüel and Kjær Type 4938, 4 Hz–70 kHz bandwidth, Norcross, GA) signal was pre-amplified (Brüel and Kjær, dual microphone supply 5935L) and high pass filtered at 10 Hz. Microphone output was calibrated to 94 dB SPL at 1 kHz prior to the measurements using an SPL calibrator (Brüel and Kjær Type 4231). The temperature was recorded with a probe (model TC 100, CWE, Inc., Ardmore, PA).

A custom written MATLAB program was used to analyze the data. The sound level recordings were analyzed using 28 1/3-octave filter bands spanning from 80 Hz to 40.3 kHz. These data give the equivalent dB SPL in each 1/3 octave band. Broadband sound levels were computed using a Z weighting (Zero frequency weighting) of the spectrum (International Standards, ICE 61672∶2003), which provides a flat frequency weighting across the sensitivity range of the sound level meter or microphone. Here, we modified the Z weighting to consider the frequencies from 1–40.3 kHz, as this range spanned from the lowest measurable frequency in the mouse audiogram (1 kHz) to the highest reliable recording from the microphone (40.3 kHz). Traditional sound level frequency weights (“A” and “C”) are not applicable to mouse hearing, as these measures predominantly weight the low frequencies over which human hearing extends. The Z-weighted broadband sound levels reported here are expected to correspond with the perceptual loudness of the environmental sounds as experienced by a mouse.

### Statistics

The statistical significance of differences for different variables was determined using a mixed effects analysis of variance [39]. Mouse was considered a random effect, while the medial or lateral glomerular location and the cage environment were considered fixed effects. The mixed ANOVA was conducted using the SAS procedure MIXED [40]. A post-hoc Tukey-Kramer test generated P values for all comparisons. Data were plotted with Microcal Origin 7.0.

## References

[1] Polley DB, Kvasnak E, Frostig RD (2004). Naturalistic experience transforms sensory maps in the adult cortex of caged animals.. Nature.

[2] Castelhano-Carlos MJ, Baumans V (2009). The impact of light, noise, cage cleaning and in-house transport on welfare and stress of laboratory rats.. Lab Anim.

[3] Gonder JC, Laber K (2007). A renewed look at laboratory rodent housing and management.. ILAR J.

[4] Mandairon N, Stack C, Kiselycznyk C, Linster C (2006). Enrichment to odors improves olfactory discrimination in adult rats.. Behav Neurosci.

[5] Lewejohann L, Reinhard C, Schrewe A, Brandewiede J, Haemisch A (2006). Environmental bias? Effects of housing conditions, laboratory environment and experimenter on behavioral tests.. Genes Brain Behav.

[6] Wahlsten D, Bachmanov A, Finn DA, Crabbe JC (2006). Stability of inbred mouse strain differences in behavior and brain size between laboratories and across decades.. Proc Natl Acad Sci U S A.

[7] Zou DJ, Feinstein P, Rivers AL, Mathews GA, Kim A (2004). Postnatal refinement of peripheral olfactory projections.. Science.

[8] Brunjes PC (1994). Unilateral naris closure and olfactory system development.. Brain Res Brain Res Rev.

[9] Mandiyan VS, Coats JK, Shah NM (2005). Deficits in sexual and aggressive behaviors in Cnga2 mutant mice.. Nat Neurosci.

[10] Kimchi T, Xu J, Dulac C (2007). A functional circuit underlying male sexual behaviour in the female mouse brain.. Nature.

[11] Hasen NS, Gammie SC (2009). Trpc2 gene impacts on maternal aggression, accessory olfactory bulb anatomy and brain activity.. Genes Brain Behav.

[12] Halpern M, Martinez-Marcos A (2003). Structure and function of the vomeronasal system: an update.. Prog Neurobiol.

[13] Shepherd GM, Chen WR, Greer CA, Shepherd GM (2004). olfactory bulb.. The Synaptic Organization of the Brain.

[14] Mombaerts P, Wang F, Dulac C, Chao SK, Nemes A (1996). Visualizing an olfactory sensory map.. Cell.

[15] Oliva AM, Jones KR, Restrepo D (2008). Sensory-dependent asymmetry for a urine-responsive olfactory bulb glomerulus.. J Comp Neurol.

[16] Restrepo D, Lin W, Salcedo E, Yamazaki K, Beauchamp G (2006). Odortypes and MHC peptides: Complementary chemosignals of MHC haplotype?. Trends Neurosci.

[17] Doty RL (1986). Odor-guided behavior in mammals.. Experientia.

[18] Brennan PA (2009). Outstanding issues surrounding vomeronasal mechanisms of pregnancy block and individual recognition in mice.. Behav Brain Res.

[19] Schaefer ML, Finger TE, Restrepo D (2001). Variability of position of the P2 glomerulus within a map of the mouse olfactory bulb.. J Comp Neurol.

[20] Royal SJ, Key B (1999). Development of P2 olfactory glomeruli in P2-internal ribosome entry site-tau-LacZ transgenic mice.. J Neurosci.

[21] Zheng C, Feinstein P, Bozza T, Rodriguez I, Mombaerts P (2000). Peripheral olfactory projections are differentially affected in mice deficient in a cyclic nucleotide-gated channel subunit.. Neuron.

[22] Lin DM, Wang F, Lowe G, Gold GH, Axel R (2000). Formation of precise connections in the olfactory bulb occurs in the absence of odorant-evoked neuronal activity.. Neuron.

[23] Goymann W, Mostl E, Gwinner E (2002). Non-invasive methods to measure androgen metabolites in excrements of European stonechats, Saxicola torquata rubicola.. Gen Comp Endocrinol.

[24] Vazquez-Palacios G, Retana-Marquez S, Bonilla-Jaime H, Velazquez-Moctezuma J (2001). Further definition of the effect of corticosterone on the sleep-wake pattern in the male rat.. Pharmacol Biochem Behav.

[25] Koay G, Heffner R, Heffner H (2002). Behavioral audiograms of homozygous med(J) mutant mice with sodium channel deficiency and unaffected controls.. Hear Res.

[26] Kryter KD (1985). The effects of noise on man..

[27] Wahlsten D, Metten P, Phillips TJ, Boehm SL, 2nd, Burkhart-Kasch S (2003). Different data from different labs: lessons from studies of gene-environment interaction.. J Neurobiol.

[28] Crabbe JC, Wahlsten D (2003). Of mice and their environments.. Science.

[29] Izidio GS, Lopes DM, Spricigo L, Ramos A (2005). Common variations in the pretest environment influence genotypic comparisons in models of anxiety.. Genes Brain Behav.

[30] Dotson CD, Spector AC (2005). Drinking spout orifice size affects licking behavior in inbred mice.. Physiol Behav.

[31] Chesler EJ, Wilson SG, Lariviere WR, Rodriguez-Zas SL, Mogil JS (2002). Identification and ranking of genetic and laboratory environment factors influencing a behavioral trait, thermal nociception, via computational analysis of a large data archive.. Neurosci Biobehav Rev.

[32] Tordoff MG, Alarcon LK, Byerly EA, Doman SA (2005). Mice acquire flavor preferences during shipping.. Physiol Behav.

[33] Heiligenberg W (1991). The neural basis of behavior: a neuroethological view.. Annu Rev Neurosci.

[34] Hoyle G (1984). The Scope of Neuroethology.. Behavioral and Brain Sciences.

[35] Watson KK, Platt ML (2008). Neuroethology of reward and decision making.. Philos Trans R Soc Lond B Biol Sci.

[36] Hurst JL, Beynon RJ (2004). Scent wars: the chemobiology of competitive signalling in mice.. Bioessays.

[37] Takahashi A, Kato K, Makino J, Shiroishi T, Koide T (2006). Multivariate analysis of temporal descriptions of open-field behavior in wild-derived mouse strains.. Behav Genet.

[38] Baker H, Morel K, Stone DM, Maruniak JA (1993). Adult naris closure profoundly reduces tyrosine hydroxylase expression in mouse olfactory bulb.. Brain Res.

[39] Searle SR, George C, Charles EM (1992).

[40] Inc SASI (2004). SAS/STATr 9.1 User's Guide..

